# Evaluation of risk factors and outcome of patients with polymyxin-resistant critical pathogens gram-negative bacilli in Hospital Kuala Lumpur

**DOI:** 10.1080/20523211.2025.2579209

**Published:** 2025-11-03

**Authors:** Rahela Ambaras Khan, Mak Woh Yon, Anitha Ramadas, Tan Sin Yee, Victor Ong Sheng Teck, Siti Nurul Asikin Makhtar, Chang Yee Shan, Leong Chee Loon, Nurzam Suhaila Che Husin, Khairil Erwan Khalid, Rohaidah Hashim, Ismaliza Ismail

**Affiliations:** a Department of Pharmacy, Hospital Kuala Lumpur, Kuala Lumpur, Malaysia; b Department of Pharmacy, Kuala Lumpur Health Clinic, Kuala Lumpur, Malaysia; c Department of Medical, Hospital Kuala Lumpur, Kuala Lumpur, Malaysia; d Department of Pathology, Hospital Kuala Lumpur, Kuala Lumpur, Malaysia; e Department of Bacteriology, Institut Penyelidikan Perubatan, Kuala Lumpur, Malaysia

**Keywords:** Polymyxin-resistant, intensive-care-unit, Malaysia, antibiotic

## Abstract

**Background:**

Polymyxin-resistant gram-negative bacilli pose a formidable challenge in clinical settings globally and the increasing prevalence of resistance has raised concerns regarding treatment options and patient outcomes. This study aimed to evaluate the risk factors, length of hospital stay and mortality among patients with polymyxin-resistant critical pathogens gram-negative bacilli (PR-CP-GNB) and polymyxin-susceptible (PS-CP-GNB) isolates.

**Methods:**

A one-year case–control study was conducted using retrospective data from patients admitted to Kuala Lumpur Hospital with a positive culture of CP-GNB. CP referred to Carbapenem-Resistant Acinetobacter baumannii and Carbapenem-Resistant Enterobacterales. Patients with PR-CP-GNB isolates were classified as cases, while those with PS-CP-GNB isolates served as controls. Mortality outcomes were assessed using Chi-Square and Fisher’s exact tests, while the length of hospital stay was evaluated using the Mann–Whitney U test. Simple and multiple logistic regression analyses were performed to identify risk factors for PR-CP-GNB.

**Results:**

The median age of the 70 patients was 55.5 (IQR 42.6-64.9), with an equal distribution between males and females. Of 70 patients, 50 (71.4%) were identified as PR-CP-GNB and the remaining were PS-CP-GNB. Additionally, 15 isolates were identified as colonisers (12 PR-CP-GNB and 3 PS-CP-GNB). Among the 55 non-coloniser, 38 (69%) were identified as PR-CP-GNB while 17 (31%) had PS-CP-GNB. No significant difference in all-cause mortality (PR-CP-GNB: 60.5%; PS-CP-GNB: 64.7%, *p* = 0.768) or infection-related mortality (PR-CP-GNB: 65.2%, PS-CP-GNB: 45.5%, *p* = 0.458) among non-coloniser patients. The median length of hospital stay post-infection was also comparable (PR-CP-GNB: 24 days vs PS-CP-GNB: 25 days, *p* = 0.791). A previous ICU admission within the last 90 days was associated with approximately six times higher odds of isolating PR-CP-GNB (AOR = 5.90, 95% CI 1.17-29.77, *p* = 0.032).

**Conclusion:**

Prior ICU admission significantly increased the risk of PR-CP-GNB isolation. Clinicians should consider a history of ICU admission as a major risk factor when evaluating patients for potential polymyxin-resistant infections.

## 1. Background

Polymyxins are a class of antibiotics comprising five chemically distinct compounds, namely polymyxins A to E. Clinically, only polymyxin B and polymyxin E (colistin) are used, which they differ by a single amino acid in their heptapeptide ring (Benedict & Langlykke, 1947). Polymyxins act by binding to the lipid A component of the lipopolysaccharide in the outer membrane of gram-negative bacteria, disrupting membrane integrity and causing cell death (Benedict & Langlykke, 1947). This unique mode of action makes polymyxins effective against multidrug-resistant gram- negative organisms which include carbapenem-resistant *Enterobacterales* (CRE), *Acinetobacter baumannii* and *Pseudomonas aeruginosa* (Li et al., 2019; Ni et al., 2015; Olaitan et al., 2014). These pathogens are common culprits of hospital-acquired infections such as bloodstream infections, pneumonia, intraabdominal infections and skin and soft tissue infections, particularly in critically ill patients. Despite their efficacy, the use of polymyxins has been increasingly compromised by the emergence of resistance, leaving limited therapeutic options for these infections (Li et al., 2019; Ni et al., 2015; Olaitan et al., 2014).

The widespread use of polymyxins in human health and animal health has significantly contributed to the rising prevalence of resistance (Cai et al., 2012; Catry et al., 2015; da Silva et al., 2020; Teo et al., 2019). In human health, polymyxins are reserved for severe infections caused by multidrug-resistant organisms, often as a last-line treatment due to limited alternatives and their associated toxicity. In veterinary and agricultural sectors, polymyxins have been employed not only to treat infections but also as growth promoters in livestock which led to the spread of resistance and global health concern (Cai et al., 2012; Catry et al., 2015; da Silva et al., 2020; Teo et al., 2019).

The increased rate of polymyxin-resistant infections has created significant challenges for clinicians as these infections are associated with limited treatment options, prolonged hospital stays and adverse outcomes (Capone et al., 2013; da Silva et al., 2020; Qamar et al., 2017; Teo et al., 2019). A few risk factors have been identified as contributors to the emergence of polymyxin resistance. These include renal failure, prolonged use of urinary catheters, frequent inter-hospital transfers, prior use of carbapenems and recent surgical procedure (da Silva et al., 2020). Understanding these risk factors is critical for implementing targeted infection prevention strategies and optimising antimicrobial stewardship programs to mitigate the emergence and spread of polymyxin-resistant gram-negative pathogens.

Studies evaluating the impact of polymyxin resistance on patient outcomes consistently indicate a higher risk of mortality, although the statistical significance varies (Capone et al., 2013; da Silva et al., 2020; Teo et al., 2019). For instance, a study in Singapore found that in-hospital mortality was higher in patients with carbapenem-resistance Enterobacterales (CRE) harbouring polymyxin resistance (50%) compared to polymyxin-sensitive CRE (38.1%). However, the difference was non statistically significant (Teo et al., 2019). Conversely, a Brazilian study reported a significantly higher mortality rate of 66.6% in patients infected with polymyxin-resistant strains compared to those with sensitive strains (da Silva et al., 2020). These findings suggest a consistent trend of increased mortality associated with polymyxin resistance but underscore the need for further research with a bigger sample size to delineate the true impact of polymyxin resistance on clinical outcomes and the significance of the association.

A local study identified key predictors of polymyxin B treatment failure in critically ill patients with healthcare-associated infections (Ismail et al., 2017). This finding highlights the limitations of current treatment options for multidrug resistant-gram negative bacilli infections and the urgency to address factors contributing to resistance and treatment failure in high-risk populations.

To date, there is a paucity of evidence on the risk factors of polymyxin-resistant gram-negative organisms in Malaysia, despite the growing global concern about resistance to this last-line antibiotic. Given the critical role of polymyxins in treating multidrug-resistant gram-negative infections and the increasing prevalence of resistance, it is essential to identify the factors driving resistance in local settings. This knowledge is crucial to inform targeted measures such as combination therapy or pharmacokinetic and pharmacodynamic optimisation, to minimise the risk of resistance and preserve the efficacy of this vital antibiotic class.

Thus, the primary objective of our study was to assess the risk factors associated with polymyxin-resistant (PR) critical pathogens (CP) gram-negative bacilli (GNB). Additionally, the study aimed to describe the clinical outcomes of patients with PR-GNB infections, including mortality and length of hospital stay. By addressing these objectives, this study seeks to contribute to the evidence necessary to guide clinical decision-making and antimicrobial stewardship efforts in managing polymyxin-resistance infections.

## 2. Methods

### 2.1 Study design

This retrospective study employed a case–control study design to identify the risk factors associated with the isolation of polymyxin-resistant critical pathogens gram-negative bacilli (PR-CP-GNB) among hospitalised adult patients to evaluate clinical outcomes which include length of hospital stay and mortality. It has been conducted in the largest hospital in Kuala Lumpur for one year period. All adult patient with positive culture of CP-GNB will be recruited into the study. Each patient was included in the study only once, at the time of the first PR/PS-CP-GNB. This study has obtained Ethical Approval from Medical Research & Ethics Committee, Malaysia [NMRR ID-22-00817-EBG (IIR)].

### 2.2 Definition

Critical pathogen is referred to Carbapenem-Resistant *Acinetobacter baumannii* (CRAB) and Carbapenem-Resistant Enterobacterales (CRE), following WHO priority pathogens classification.

Cases were defined as patients with PR-GNB isolates from a clinical sample namely blood, tracheal aspirate or urine, while controls were patients with polymyxin-susceptible critical pathogens gram-negative bacilli (PS-CP-GNB) from similar samples.

Polymyxin susceptibility is adhered to Clinical and Laboratory Standards Institute (CLSI) breakpoint for the susceptible interpretive category during data collection period. Antimicrobial Susceptibility Testing (AST) for polymyxins is not performed routinely on-site and is referred to the Institute for Medical Research (IMR), Malaysia which serves as the national reference laboratory. The typical turnaround time for final polymyxin AST results from IMR is approximately 7–14 days.

### 2.3 Data collection

Data were extracted retrospectively from medical records and electronic microbiology reports, focusing on patient demographic, comorbidities, history of hospitalisations or ICU stays, invasive medical device use, previous antibiotic exposure within 30 days, microbiological characteristic and outcomes. Outcome data include all-cause mortality and length of hospital stay.

### 2.4 Data analysis

Categorical variables were analysed using the chi-square test or Fisher’s exact test (when cell counts were <5). Continuous variables were tested for normality and comparisons were made using the Mann–Whitney U test for non-parametric data.

Risk factors for PR-GNB were analysed using logistic regression. Variables with *p*-values <0.20 in univariate analysis or those deemed clinically relevant were included in a multivariable logistic regression model.

All cause-mortality and infection-related mortality were compared between cases and controls using chi-square or Fisher’s exact tests. Differences in the length of hospital stay were analysed using Mann–Whitney U test due to non-normal distribution of data.

A *p*-value <0.05 was considered statistically significant for all analyses. Results were reported as odd ratios (OR) with 95% confidence intervals (CI) for logistic regression.

## 3. Results

### 3.1 Patient characteristic

A total of 70 patients were included in the study, comprising both PR-CP-GNB and PS-CP-GNB groups. The median age of the overall population was 55.5 years (IQR: 42.6-64.9), with no significant difference between the PR-CP-GNB and PS-CP-GNB groups (*p* = 0.394). Gender distribution was approximately equal, with males comprising 48% of the PR-CP-GNB group and 55% of the PS-CP-GNB group (*p* = 0.597). Ethnic composition was similar between the groups, with Malay patients representing the majority in both PR-CP-GNB (58%) and PS-CP-GNB (65%, *p* = 0.787). In short, there are no significant demographic differences namely age, gender, or ethnicity between PR-CP-GNB and PS-CP-GNB (Table 1).

**Table 1.** Patient characteristics of the study population (N = 70).

	Total	PR-GNB (nn = 50)	PS-GNB (nn = 20)	*p*-value
	n (%)	n (%)	n (%)	
Age (years)				
Median (IQR)	55.5 (42.6-64.9)	51.7 (35.8-65.4)	56.9 (52.0-63.0)	0.394^a^
Gender				
Male	35 (50.0)	24 (48.0)	11 (55.0)	0.597
Female	35 (50.0)	26 (52.0)	9 (45.0)	
Race				
Malay	42 (60.0)	29 (58.0)	13 (65.0)	0.787^b^
Chinese	11 (15.7)	8 (16.0)	3 (15.0)	
Indian	9 (12.9)	6 (12.0)	3 (15.0)	
Others	8 (11.4)	7 (14.0)	1 (5.0)	
Medical history				
Cancer/malignancy	4 (5.7)	3 (6.0)	1 (5.0)	1.000^b^
Diabetes mellitus	31 (44.3)	23 (46.0)	8 (40.0)	0.648
Renal disease	13 (18.6)	10 (20.0)	3 (15.0)	0.744^b^
Receipt of steroid	3 (4.3)	2 (4.0)	1 (5.0)	1.000^b^
Recent surgery/invasive procedure	26 (37.1)	19 (38.0)	7 (35.0)	0.814
Previous contact with healthcare facilities^c^				
	33 (47.1)	25 (50.0)	8 (40.0)	0.449
Previous admission to ICU^d^	19 (27.1)	17 (34.0)	2 (10.0)	0.041*
Type of medical devices used in the last 30 days				
Arterial catheter	15 (21.4)	12 (24.0)	3 (15.0)	0.528
Central venous catheter	39 (55.7)	25 (50.0)	14 (70.0)	0.128
Mechanical ventilation	33 (47.1)	25 (50.0)	8 (40.0)	1.000^b^
Peripheral venous catheter	51 (72.9)	38 (76.0)	13 (65.0)	0.35
Tracheostomy	14 (20.0)	11 (22.0)	3 (15.0)	0.449
Urinary catheter	53 (75.7)	38 (76.0)	15 (75.0)	0.742^b^
Antibiotic exposure^c^				
Carbapenem	21 (30.0)	16 (32.0)	5 (25.0)	0.564
Polymyxin	5 (7.1)	4 (8.0)	1 (5.0)	1.000^b^
Third generation cephalosporin	43 (61.4)	31 (62.0)	12 (60.0)	0.877
Beta lactam/beta-lactamase inhibitor	24 (34.3)	16 (32.0)	8 (40.0)	0.524

Chi-square test was performed unless otherwise stated.

^a^ Mann-Whitney U test was performed. Data is not normally distributed.

^b^ Fisher’s exact test was performed as more than 20% of cells have expected count less than 5.

^c^ Within the last 30 days prior to the day of MDRO culture obtained.

^d^ Within the last 90 days prior to the day of MDRO culture obtained.

*p < 0.05 denotes statistical significance.

Higher proportion of prior ICU admissions in the PR-CP-GNB group (34%) compared to the PS-CP-GNB (10%, *p* = 0.041). This highlights ICU admission as a critical factor for PR-CP-GNB, likely due to invasive interventions and antibiotic use in critically ill patients (Table 1).

Other evaluated factors, which include comorbidities such as diabetes, renal disease and cancer, were prevalent but not significantly associated with PR-GNB. Healthcare facilities exposure and recent invasive procedures showed no significant differences between PR-CP-GNB and PS-CP-GNB groups, suggesting these variables alone may not independently contribute to the development of resistant organism (Table 1).

The use of invasive medical devices was commonly used in both groups, with urinary catheters (76% in PR-CP-GNB vs 75% in PS-CP-GNB, *p* = 0.742) and peripheral venous catheters (76% vs 65%, *p* = 0.350) being the most frequently used. Central venous catheters were more common in the PS-CP-GNB (70% vs 50%, *p* = 0.128), although this difference was not statistically significant. Antibiotic exposure within the last 30 days, including carbapenem (32% vs 25%, *p* = 0.564) and polymyxins (8% vs 5%, *p* = 1.000), was also similar between groups, reflecting broad spectrum antibiotic usage in this study population (Table 1).

### 3.2 Microorganisms

*Acinetobacter spp.* was the predominant species in both groups, comprising 74% of PR-CP-GNB isolates and 65% PS-CP-GNB isolates, with no significant differences in the distribution of microorganism species (*p* = 0.399). Other species including Enterobacter spp., *E. coli and K. pneumonia* were less commonly isolated in both groups (Table 2).

**Table 2.** Microbiological characteristics (N = 70)

Characteristics	Total	PR-GNB (nn = 50)	PS-GNB (nn = 20)	*p*-value
	n (%)	n (%)	n (%)	
Species of microorganism isolated				
*Acinetobacter spp.*	50 (71.4)	37 (74.0)	13 (65.0)	0.399^a^
*Enterobacter spp.*	4 (5.7)	2 (4.0)	2 (10.0)	
*Escherichia coli*	3 (4.3)	3 (6.0)	0 (0.0)	
*Klebsiella pneumoniae*	13 (18.6)	8 (16.0)	5 (25.0)	
Specimen type				
Blood	37 (52.9)	25 (50.0)	12 (60.0)	0.787^a^
CSF	11 (15.7)	9 (18.0)	2 (10.0)	
Respiratory	1 (1.4)	1 (2.0)	0 (0.0)	
Skin and soft tissue	15 (21.4)	10 (20.0)	5 (25.0)	
Urine	1 (1.4)	1 (2.0)	0 (0.0)	
Others	5 (7.1)	4 (8.0)	1 (5.0)	

Chi-square test was performed unless otherwise stated.

^a^ Fisher’s exact test was performed as more than 20% of cells have expected count less than 5.

*p < 0.05 denotes statistical significance.

Regarding specimen types, blood was the most frequent source isolated, accounting 50% in the PR-CP-GNB and 60% in PS-CP-GNB, followed by skin and soft tissue and cerebrospinal fluid samples. There were no significant differences in specimen types between the groups (*p* = 0.787) (Table 2).

### 3.3 Risk factors associated with polymyxin-resistant gram-negative bacilli

In univariate analysis, previous ICU admission emerged as a strong predictor for PR-CP-GNB, with an unadjusted odds ratio (OR) of 4.64 (95% CI: 0.96-22.37, *p* = 0.056). In multivariable analysis, ICU admission remained significant with an adjusted OR of 5.9 (95% CI: 1.17-29.77, *p* = 0.032). Central venous catheter use showed a borderline association (adjusted OR = 0.33, 95% CI: 0.10-1.05, *p* = 0.062), although it is not statistically significant (Table 3).

**Table 3.** Risk factors predicting isolation of Polymyxin-Resistant Critical Pathogens Gram-Negative Bacilli (PR-CP-GNB)

Variables	Simple logistic regression		Multiple logistic regression^e^	
	Unadjusted OR (95% CI)	*p* value	Adjusted OR	*p* value
			(95% CI)	
Age	0.98 (0.95, 1.02)	0.323		
Gender:				
Male	1	0.706		
Female	1.22 (0.43, 3.46)			
Ethnicity:				
Malay	1	0.813		
Chinese	1.20 (0.27, 5.25)	0.889		
Indian	0.90 (0.19, 4.15)	0.307		
Others	3.14 (0.25, 28.18)			
Medical history:				
Diabetes mellitus	1.28 (0.45, 3.66)	0.648		
Renal disease	1.42 (0.35, 5.80)	0.628		
Cancer/malignancy	1.21 (0.12, 12.40)	0.871		
Receipt of steroid	0.79 (0.07, 9.25)	0.852		
Recent surgery/invasive procedure	1.14 (0.39, 3.36)	0.815		
Previous contact with healthcare facilities in the last 30 days	1.50 (0.52, 4.30)	0.450		
Previous ICU admission^a^	4.64 (0.96, 22.37)	0.056	5.90 (1.17, 29.77)	0.032*
Types of medical devices used:				
Urinary catheter use	1.06 (0.32, 3.51)	0.930		
Mechanical ventilation	1.50 (0.52, 4.30)	0.450		
Tracheostomy	1.60 (0.40, 6.47)	0.511		
Central venous catheter^a^	0.43 (1.42, 1.29)	0.133	0.33 (0.10, 1.05)	0.062
Arterial line	1.79 (0.45, 7.17)	0.411		
Peripheral line	1.71 (0.55, 5.25)	0.352		
Antibiotic exposure				
Carbapenem	1.41 (0.44, 4.57)	0.565		
Polymyxin	1.65 (1.17, 15.76)	0.663		
Third generation cephalosporin	1.09 (0.38, 3.14)	0.877		
Beta lactam/beta-lactamase inhibitor	0.71 (0.24, 2.07)	0.525		

^a^ Variables included in the multiple logistic regression.

OR: odds ratio.

CI: confidence interval.

*p < 0.05 denotes statistical significance.

^e^ Backward LR selection method applied.

No interactions and multicollinearity detected.

Hosmer-Lemeshow test, *p* = 0.824.

Classification table 71.4% correctly classified.

Area under ROC curve = 0.698 (95% CI: 0.57-0.83, *p* = 0.010).

Other factors, including age, gender, comorbidities, previous healthcare exposure and recent antibiotic use were not significantly associated with PR-CP-GNB (Table 3).

### 3.4 Length of stay and mortality outcomes

Among 55 patients with confirmed infections, the overall mortality rate was 61.8%. All-cause mortality was similar between patients with PR-CP-GNB (60.5%) and PS-CP-GNB (64.7%) with no statistically significant difference (*p* = 0.768) (Table 4). Infection-related mortality rates were also comparable, at 65.2% for PR-CP-GNB and 45.5% for PS-CP-GNB (*p* = 0.458). These findings suggest that the presence of polymyxin resistance does not significantly influence mortality, with high fatality rates likely reflecting the severity of illness in the study population.

**Table 4.** Outcomes of patients with Polymyxin-Resistant Critical Pathogens Gram-Negative Bacilli (PR-CP-GNB) and Polymyxin-Susceptible Critical Pathogens Gram-Negative Bacilli (PS-CP-GNB) (N = 55).

Outcomes	Total	PR-CP-GNB (n = 38)	PS-CP-GNB (n = 17)	*p*-value
	n (%)	n (%)	n (%)	
All-cause mortality	34 (61.8)	23 (60.5)	11 (64.7)	0.768
Infection-related mortality	20 (58.8)	15 (65.2)	5 (45.5)	0.458^a^
Length of hospital stay post infection (days)^c^:				
Median (IQR)	24 (14.5, 38.5)	24 (14.0, 36.0)	25 (13.5, 47.75)	0.791^b^

Chi-square test was performed unless otherwise stated.

^a^ Fisher’s exact test was performed as more than 20% of cells have expected count less than 5.

^b^ Mann-Whitney U test was performed (non-normally distributed).

^c^ Only consider alive patients.

*p < 0.05 denotes statistical significance.

The median length of hospital stay post-infection was prolonged for both groups, with PR-CP-GNB patients staying a median of 24 days (IQR:14.0-36.0) compared to 25 days (IQR: 13.5-47.8) for PS-CP-GNB patients (Table 4). However, there was no significant difference between the two groups (*p* = 0.791) (Table 4). These results indicate that infections caused by both PR-CP-GNB and PS-CP-GNB lead to significant healthcare burdens, regardless of polymyxin susceptibility.

## 4. Discussion

This study was conducted to address the growing concern of polymyxin resistance among GNB, a critical challenge in managing multidrug-resistant infections. Despite the increasing use of polymyxins as last-line antibiotics, evidence on the risk factor contributing to resistance and the clinical outcomes associated with PR-CP-GNB remains limited, particularly in Malaysia and Low- and Middle-Income Countries (LMICs). Understanding these factors is essential for guiding antimicrobial stewardship practices, improving patient outcomes and developing targeted strategies to mitigate resistance.

In the current study, prior ICU admission was identified as a significant independent risk factor for PR-CP-GNB, reflecting the vulnerability of critically ill patients who are frequently exposed to invasive procedure, broad-spectrum antibiotics and prolonged hospital stays. This study aligns with previous studies that have highlighted ICU settings as critical environments for the development for antimicrobial resistance due to extensive antibiotic use (da Silva et al., 2020; Teo et al., 2019). Hence, optimising dosing strategies, adhering to pharmacokinetic and pharmacodynamic principles and considering combination therapy is essential to improve patient outcomes (Qamar et al., 2017).

No significant differences in mortality were observed between PR-CP-GNB and PS-CP-GNB group in this study contrasting those studies from Italy (Capone et al., 2013), Brazil (da Silva et al., 2020) and Singapore (Teo et al., 2019) which reported increased mortality in PR-GNB infections, though with varying levels of statistical significance. The differences in mortality outcomes between this study and others could be attributed to several factors, including smaller sample size in this study which may limit its statistical power to detect significant differences. Variation in patient populations such as different comorbidity profile and severity of illness could also influence the results. The predominant pathogen in each study and its associated virulence may further contribute to discrepancies, as well as whether studies included colonised or focused solely on infections.

The findings of a local study complement this study by highlighting the challenges of managing infections caused by multidrug-resistant gram-negative organisms in critically ill patients particularly regarding the efficacy of polymyxin B therapy (Ismail et al., 2017). While this study identifies ICU admission as a significant risk factor isolating PR-CP-GNB, that local study focuses on treatment failure predictors, emphasising the role of appropriate dosing strategies to optimise outcomes. Both studies underscore the burden of multidrug-resistant GNB infections in ICU settings and the limitation of current therapeutic options that may lead to higher failure rates. Together, these findings highlight the need for targeted interventions such as optimising drug selection and dosing to improve outcomes of critically ill patient with resistant infections.

Central venous catheter use showed a borderline association with the development of PS-CP-GNB, though was not statistically significant. This indicates that while this factor is prevalent in the study population, they may contribute to multidrug resistance broadly rather than being specific to polymyxin resistance. This result emphasises the multifaceted nature of PR-CP-GNB acquisition, and the urgent need for ICU-targeted strategies and robust antimicrobial stewardship to mitigate resistance.

While this study provides valuable insights into the risk factors and outcomes associated with PR-CP-GNB, several limitations must be acknowledged. The retrospective design and relatively small sample size may limit the generalizability of the findings and the statistical power to detect associations for less common risk factors. Additionally, the absence of molecular data on resistance mechanism limits the ability to explore specific genetic contributors to polymyxin resistance. Future studies should aim to incorporate larger, multicentre cohorts and genomic analyses to better understand the drivers of resistance to identify potential targets for intervention.

## 5. Conclusion

In conclusion, this study highlight that a history of recent ICU admission significantly increases the risk of isolating PR-CP-GNB. Clinicians managing patients in the ICU should maintain a high suspicion for polymyxin resistance in those with recent ICU admission, particularly when initiating empiric antibiotic therapy, ensuring their use is guided by risk stratification, local resistance patterns and microbiological evidence. Clinicians may also incorporate alternative therapies or combination therapy while waiting for susceptibility results. This proactive approach can help to optimise treatment outcomes and minimise the inappropriate use of polymyxins, thereby reducing the risk of further resistance development.
